# Influenza Vaccine Hesitancy in Patients with Multiple Sclerosis: A Monocentric Observational Study

**DOI:** 10.3390/brainsci11070890

**Published:** 2021-07-05

**Authors:** Antonio Ziello, Cristina Scavone, Maria Elena Di Battista, Simona Salvatore, Daniele Di Giulio Cesare, Ornella Moreggia, Lia Allegorico, Anna Sagnelli, Stefano Barbato, Valentino Manzo, Annalisa Capuano, Giorgia Teresa Maniscalco

**Affiliations:** 1 Multiple Sclerosis Center “A. Cardarelli” Hospital, 80131 Naples, Italy; antonioziello@libero.it (A.Z.); elena.dibattista@aocardarelli.it (M.E.D.B.); simona.salvatore@aocardarelli.it (S.S.); daniel.dgc@alice.it (D.D.G.C.); ornellamoreggia@gmail.com (O.M.); lia.allegorico@hotmail.it (L.A.); 2 Department of Experimental Medicine, University of Campania “Luigi Vanvitelli”, 80138 Naples, Italy; cristina.scavone@unicampania.it (C.S.); annalisa.capuano@unicampania.it (A.C.); 3 Regional Center of Pharmacovigilance and Pharmacoepidemiology of Campania Region, 80138 Naples, Italy; 4 Neurological Clinic and Stroke Unit “A. Cardarelli” Hospital, 80131 Naples, Italy; anna.sagnelli@aocardarelli.it (A.S.); stefano.barbato@aocardarelli.it (S.B.); valentino.manzo@aocardarelli.it (V.M.)

**Keywords:** influenza vaccine, hesitancy, multiple sclerosis, observational study

## Abstract

Background. The so-called “vaccine hesitancy” still represents a common phenomenon that undermines the effectiveness of vaccination campaigns. In 2020, the Italian Medicines Agency recommended to bring forward the flu vaccination campaign, whose importance was also emphasized for patients with Multiple Sclerosis (MS). We aimed to assess vaccination behavior in patients with MS to prepare for the upcoming SARS-CoV-2 vaccination challenge. Methods. This is an observational study carried out in one MS clinical Centre that enrolled all MS patients who were eligible for any of the flu vaccines recommended by the Italian medicines Agency. Results. 194 patients were enrolled. Patients’ mean age was 43.9 years and 66% were female. Comorbidities, mainly represented by non-autoimmune diseases, were identified in 52% of patients. Almost all patients were receiving a DMT during the study period, mainly dimethyl fumarate, natalizumab, teriflunomide, and interferon. Out of 194 patients, 58.2% accepted to be vaccinated. No statistically significant differences were found, except for the use of natalizumab, which was higher among vaccinated patients. Conclusion. The results of our study emphasize the importance of education and communication campaigns addressed both to healthcare providers and patients with MS, especially considering that MS patients are currently receiving COVID-19 vaccinations.

## 1. Introduction

Being a chronic autoimmune disease, multiple sclerosis (MS) requires immunosuppressive or immunomodulating treatments to modifying disease course (Disease Modifying Therapies—DMTs). Consequently, MS patients taking DMTs may be at a higher risk for general viral and bacterial infections and infection-related events [1]. Until recently, MS patients were discouraged from vaccinating, as some studies found a positive association between vaccines and MS onset or relapse risk [2,3]. Currently, the safety and efficacy of vaccines against various pathogens are well established, including among MS patients [4,5] and several studies showed that there is a good response to vaccines in MS patients, which is comparable to that observed in healthy subjects [6,7]. This year, given the epidemiological situation related to the spread of SARS-CoV-2 infection, the Italian Medicines Agency (AIFA) recommended to bring forward the flu vaccination campaign, whose importance was specifically emphasized for high-risk patients (including subjects with congenital or acquired diseases, drug-induced immunosuppression, and/or chronic inflammatory diseases). Indeed, the ongoing pandemic has posed a greater risk of morbidity and mortality for immunosuppressed subjects [1]. For these reasons, in September 2020, the Italian Ministry of Health has made a further call to early vaccination of MS patients for influenza and pneumococcus [8], with the aim to build herd immunity as soon as possible. To assist physicians with decision making regarding vaccine administration in MS patients, a Delphi consensus process was undertaken to develop clinically relevant recommendations for vaccinations [9].

Despite being recognized as one of the most successful public health preventive measures, vaccinations are still perceived as unsafe and unnecessary by a growing number of individuals [10]. The “vaccine hesitancy” refers to a delay in the acceptance or refusal of vaccination, despite the availability of vaccination services. Vaccine hesitancy is complex and context-specific, varying across time, place, and vaccines [11]. This definition suggests that barriers to vaccine uptake can be very different in kind and significance.

The influenza vaccination is required annually and, in most countries, it is recommended for specific risk groups. Using flu vaccination as a model, we aimed to assess vaccination behavior in patients with MS to prepare for the upcoming SARS-CoV-2 vaccination challenge.

## 2. Results

We identified 266 MS patients; of these, 72 were excluded because of missing clinical information regarding vaccination status, resulting in a cohort of 194 patients. Overall, patients’ median age was 44 years and 66% were female. Almost 58% of patients accepted to be vaccinated (Table 1). Relapsing-Remitting Multiple Sclerosis (RRSM) was the most common form of MS, which was identified in more than 90% of patients (n = 179), while almost 7% of patients were diagnosed with Secondary Progressive Multiple Sclerosis (SPSM) and 0.5% with Primary Progressive Multiple Sclerosis (PPSM). Enrolled patients had a moderate degree of disability (mean EDSS 1.7, range 0–8.5). Approximately 52% of patients had at least one comorbidity; among concomitant diseases, non-autoimmune comorbidities were the most commonly identified (44% of patients) (Table 1). The majority of enrolled patients (98.5%) received a DMT during the study period. Among the DMTs, those prescribed in more than 10% of patients were dimethyl fumarate (23.2%), natalizumab (19.1%), teriflunomide (15.5%), and interferon (15% of enrolled patients). Lastly, almost 90% of patients had not experienced a relapse before the flu vaccination (Table 1).

The above described sociodemographic and clinical variables for vaccinated and unvaccinated groups are summarized in Table 2. Flu vaccination was performed in 113/194 (58.2%) of patients and 81/194 (41.8%) patients refused the vaccination. All patients who refused to be vaccinated declared that this choice was related to the fear of serious side effects but also the belief that the flu is not harmful. By comparing demographic and clinical characteristics between vaccinated and unvaccinated patients, no statistically significant differences were found, except for the use of natalizumab, which was higher among vaccinated patients vs. unvaccinated (11.1% vs. 24.8%, *p* = 0.01) (Table 2).

## 3. Discussion

Vaccination coverage is a growing concern in Italy. For instance, among the pediatric population, vaccine hesitancy is a widespread phenomenon that mainly derives from patients’ personal feelings and attitudes related to vaccines which, in turn, are connected with the success of vaccination itself. Indeed, the almost complete disappearance of some diseases, thanks to the development of effective vaccines, reduced the perception of the contagion’s danger and it facilitated the spread of movements opposed to vaccinations, for ethical or religious reasons, or for fear of vaccines-induced adverse events [12,13]. Similarly, the coverage for influenza vaccines in the elderly have not reached the recommended thresholds, especially after the “Fluad episode” during the influenza vaccination campaign 2014–2015 [14,15]. Indeed, the reporting of the three deaths that occurred 48 h after the vaccination with Fluad, a flu shot for adults over 65, led to the withdrawal of two batches of this vaccine. This situation sparked a great alarm, increased the fear of Fluad-induced deaths and was followed by the reporting of a total of 13 cases of death among 8 Italian regions. On December 2014, the Pharmacovigilance Risk Assessment Committee of the European Medicines Agency concluded that there was no evidence that Fluad had caused the reported deaths. This is a shining example to what extent negative feelings concerning vaccine-induced AEFIs could reduce public trust in immunizations [16,17,18]. The lack of confidence in vaccines is considered a threat to the success of vaccination programs. Old beliefs, especially those related to the safety profile of vaccines, are still rooted in Italy and it is neither simple nor immediately possible to counteract the so-called vaccine hesitancy [19]. On the other hand, the current epidemiological situation related to COVID-19 may have a significant sociopsychological influence on vaccination’s acceptance among people whether having certain diseases or not. Indeed, recent studies describing COVID-19 vaccination’s willingness in patients with MS [20,21,22] reported that almost 60–80% of interviewed MS participants were willing to receive a COVID-19 vaccine. As authors reported, vaccine willingness was statistically significant associated with increased perceived personal risk of COVID-19, prior influenza vaccine acceptance, higher educational level, older age, and discussion of the importance of COVID-19 vaccine with neurologists. Thus, a higher confidence in COVID-19 vaccines seems to exist. In our opinion, this could be probably the result of a higher perceived personal risk of COVID-19 and its seriousness compared to the perceived personal risk of common flu.

In our study, we found that the mean age of enrolled patients was equal to 43.9 years and 66% were female. RRSM was the most common form of MS, while the most commonly prescribed DMTs were dimethyl fumarate, natalizumab, teriflunomide, and interferon. These demographic characteristics were expected. Regarding the most commonly prescribed DMTs, most of them, such as dimethyl fumarate, teriflunomide and interferon, are first-line therapies, while natalizumab is a second line therapy for MS [23]. In our opinion the distribution in DMTs’ utilization is probably related to the variegate population included in our study mainly in terms of age (the range is from 18 to 88 years old) and MS type.

Our results related to vaccine hesitancy demonstrated that 41.8% of patients refused to be vaccinated despite the availability of vaccination services. These patients were older and had less frequently an autoimmune comorbidity compared to the “vaccinated” group (even though these differences were not statistically significant). This finding may be related to older people’s behavior towards vaccination and how advanced age can lead to vaccine hesitancy [24]. The percentage of patients who refused the vaccination in our study is very similar to that reported in another observational study. Indeed, Diem et al. recently carried out a retrospective study with the aim to evaluate the behavior of MS patients with regards to pneumococcal vaccination. Their findings highlighted that almost 40% of patients refused to be vaccinated. However, contrary to our results, Diem et al. found out that compared to vaccinated patients those who refused vaccination were younger. In addition, unvaccinated patients were more commonly diagnosed with psychiatric diseases [25].

From our study, interesting data came from the evaluation of DMTs, as patients treated with natalizumab showed greater compliance with vaccination, probably due to higher confidence in clinicians of MS center, resulting from the greater frequency of clinical evaluations of these patients. In fact, the confidence in the healthcare system and in clinicians (both neurologists and general practitioners), who inform patients about vaccines, plays an important role in the decision to be vaccinated [26]. The vaccine misinformation spread by major social media companies is a common problem, especially in the light of the newly available COVID-19 vaccines [27]. In addition, one of the main concerns related to vaccination in MS patients is related to a variable vaccination response according to ongoing DMTs treatment. Indeed, a study by Ciotti Jr et al. revealed that the response to vaccines seems to be reduced among patients receiving glatiramer acetate, teriflunomide, sphingosine-1-phosphate receptor modulators, and natalizumab. This effect is thought to be related to humoral vaccine responses that can be significantly impaired by B cell depleting anti-CD20 monoclonal antibody therapies. However, as some authors highlighted, the responses to vaccinations depend on many other different factors, including the vaccine type and the impact of DMTs on humoral and cellular immunity. When considering a given therapy, clinicians should weigh its efficacy against MS for the individual patient versus potential impact on responses to vaccinations that may be needed in the future [28].

As previously reported, vaccine hesitancy still represents a problem for global healthcare systems. The reduced awareness of diseases’ severity and the lack of confidence in healthcare authorities undoubtedly represent the most important barriers to vaccine uptake, especially in older people facing the chronic disease [29]. The absence of specific autoimmune comorbidities in our population may have exacerbated this aspect. Moreover, individuals who refused to be vaccinated often share a particular worldview regarding health (e.g., a preference for natural medicine and the belief that good hygiene and personal habits can make vaccination unnecessary) [29]. Similarly, the increasing popularity of complementary and alternative medicine plays an important role in the vaccination skepticism of the population [25].

Therefore, vaccinations’ importance should be highlighted especially among patients with MS. Indeed, it is well known that many common viruses, including Influenza A, Epstein-Barr, and herpesviruses, can cause MS relapses [30,31,32]. In addition, an increase in MS relapse was found to coincide with the occurrence of the common cold, of which 50–75% is thought to originate from rhinoviral infection of the upper respiratory system [33]. Many mechanisms seem to underline the association between viral infections and MS relapses, including: the activation of heterologous T-cells resulting from the viral infection with TCR specificity for self-epitopes; the release of superantigen that bind Vβ regions of the T-cell receptor (TCR) and MHC class II, resulting in T-cell activation; the release of self-antigens used to prime self-reactive T-cells resulting from chronic inflammation [34].

Based on these considerations and given that vaccinations might not represent a risk factor for MS relapses, a better vaccines education should be promoted among patients with MS in order to achieve higher vaccination coverage and, consequently, protection against infections.

## 4. Methods

### 4.1. Study Design

This was a prospective observational study, carried out from October 2020 to April 2021 among the MS Centre of the Cardarelli Hospital (Naples, Italy). The study enrolled all MS patients who were eligible for any of the flu vaccines recommended by the AIFA [8].

In order to evaluate the study’s objective, enrolled MS patients, according to their personal decision on whether to be vaccinated or not, were classified into the following two groups: the “vaccinated” group (those patients who accepted the flu vaccine) and the “unvaccinated” group (those patients who refused the flu vaccine).

### 4.2. Demographic and Clinical Data Collection

The following variables were extracted from medical records: age, sex, Expanded Disability Status Scale (EDSS), MS diagnosis according to the 2010 McDonald criteria [35], type of MS, DMTs, clinical relapse before vaccination (up 12 months), and concomitant disease. Reasons for vaccination’s refusal were collected as well. Patients’ general practitioners were informed about the methods and aims of the project and they played a key role within the study since they provided information on the flu vaccine administered.

### 4.3. Data Analysis

Descriptive statistics were used to summarize variables. The comparison between the two groups (vaccinated vs. unvaccinated) was evaluated by a chi square test and fisher’s exact test (for small samples, n < 40). A t-test for independent samples was used to compare the mean of two groups of patients and a Kruskal–Wallis non parametric test was performed for the comparison of median age; a p-value less than 0.05 was considered statistically significant.

### 4.4. Ethical Aspect

The study was approved by the Ethic Committee of the Cardarelli Hospital. Patients were informed about the methods and aims of the study and agreed to participate. A written informed consent was obtained from patients.

## 5. Study Limitations and Strengths

Our findings regarding vaccine hesitancy in a cohort of MS patients need to be considered exploratory due to many reasons. First of all, the small sample size limited our power to draw firm conclusions on the evaluated objective. Second, more than 70 patients were excluded from our study because of missing information on vaccination status. This could have introduced biases in the evaluation of the results.

Notwithstanding these limitations, to our knowledge, this is the first study carried out in Italy to evaluate vaccination attitudes among MS patients. This is a new area of clinical investigation, considering that until recently vaccinations were not recommended for MS patients. In addition, we believe that our results should be analyzed in a broader sense, especially considering the historic pandemic moment we are going through and issues related to COVID-19 vaccinations that could potentially emerge among MS patients.

## 6. Conclusions

The results of our study highlight that clear and targeted education about vaccination is imperative, also taking into account social media. Now more than ever, the acceptance of vaccines is essential in order to achieve herd immunity and to return to a normal life. Therefore, in order to increase awareness of and confidence in all vaccinations, including COVID-19 ones, there is an urgent need of vaccination campaigns that involve neurologists, general practitioners, and patients. These campaigns—based on a transparent, clear, and evidence-based communication—should be focused on the importance of vaccines, their beneficial effects, and their benefit/risk ratio. Further studies investigating the vaccination behavior in patients with MS are warranted, especially in the light of the current pandemic. Considering that the clinical course of MS could be complicated by infections that can lead to a potential increased risk of complications, prevention represents a fundamental tool to reduce this risk and to avoid interruption of MS therapy in case of serious infections.

## Figures and Tables

**Table 1:** Total patient characteristics concerning flu vaccination status.

Variable	All Patients (n = 194)
**Female n (%)**	128 (66)
**Age (y), median**	44
**Vaccinated patients n (%)**	113 (58.2)
**MS type, n (%)**	
RRMS, n (%)	179 (92.3)
SPMS, n (%)	14 (7.2)
PPMS, n (%)	1 (0.5)
**EDSS, mean (range)**	1.7 (0–8.5)
**Total comorbidity n (%)**	101 (52)
Autoimmune comorbidity n, (%) *	15 (7.7)
Non-autoimmune comorbidity n/N, (%)	86 (44.3)
**DMT for SM, n (%)**	
Interferon	29 (15)
Ocrelizumab	16 (8.3)
Glatiramer acetate	9 (4.6)
Fingolimod	14 (7.2)
Cladribrine	9 (4.6)
Dimethyl fumarate	45 (23.2)
Natalizumab	37 (19.1)
Teriflunomide	30 (15.5)
Azathioprine	1 (0.5)
Alemtuzumab	1 (0.5)
**No therapy**	3 (1.5)
**Patients with relapse before vaccination** **(up 12 months)**	
YES	19 (9.8)
NO	175 (90.2)

MS: Multiple Sclerosis; RRMS: Relapsing Remitting Multiple Sclerosis; SPMS: Secondary Progressive Multiple Sclerosis; PPMS: Primary Progressive Multiple Sclerosis; EDSS: Expanded Disability Status Scale; DMT: disease modifying therapy. *Autoimmune comorbidities: thyroiditis, psoriasis, uveitis.

**Table 2:** The sample divided in a vaccinated and unvaccinated group.

Variable	Vaccinated (n = 113)	Unvaccinated (n = 81)	*p*-Value
**Female n, (%)**	74 (65.5)	54 (66.7)	0.86
**Age (y), mean**	43	45	0.25
**EDSS, mean (range)**	1.6 (0–8.5)	1.9 (0–7.5)	0.39
**MS type**			
RRMS, n (%)	105 (92.9)	74 (91.4)	0.69
PPMS, n (%)	1 (0.9)	-	0.39
SPMS, n (%)	7 (6.2)	7 (8.6)	0.52
**Total comorbidities, n (%)**	61 (54)	40 (49.4)	0.53
Autoimmune comorbidity n, (%)	12 (19.7)	3 (7.5)	0.07
Non-autoimmune comorbidity n, (%)	49 (80.3)	37 (92.5)	0.75
**DMT for MS n, (%)**			
Interferon	13 (11.5)	16 (19.8)	0.07
Ocrelizumab	9 (8)	7 (8.6)	0.87
Glatiramer acetate	3 (2.6)	6 (7.4)	0.12
Fingolimod	8 (7.1)	6 (7.4)	0.93
Cladribrine	4 (3.5)	5 (6.2)	0.38
Dimethyl fumarate	28 (24.8)	17 (21)	0.54
Natalizumab	28 (24.8)	9 (11.1)	0.01
Teriflunomide	17 (15)	13 (16)	0.85
Azathioprine	1 (0.9)	-	0.39
Alemtuzumab	1 (0.9)	-	0.39
**No therapy**	1 (0.9)	2 (2.5)	0.38
**Patients with Relapse before vaccination** **(up to 12 months)**			
YES	13 (11.5)	6 (7.4)	0.34
NO	100 (88.5)	75 (92.6)	

MS: Multiple Sclerosis; RRMS: Relapsing Remitting Multiple Sclerosis; SPMS: Secondary Progressive Multiple Sclerosis; PPMS: Primary Progressive Multiple Sclerosis; EDSS: Expanded Disability Status Scale; DMT: disease modifying therapy.
